# Angiogenic signaling in the lungs of a metabolically suppressed hibernating mammal (*Ictidomys tridecemlineatus*)

**DOI:** 10.7717/peerj.8116

**Published:** 2019-11-18

**Authors:** Samantha M. Logan, Kenneth B. Storey

**Affiliations:** Institute of Biochemistry, Departments of Biology and Chemistry, Carleton University, Ottawa, ON, Canada

**Keywords:** VEGF, BMP-9, Soluble receptor, Metabolic suppression, Lung, Ground squirrel, Tissue remodeling

## Abstract

To conserve energy in times of limited resource availability, particularly during cold winters, hibernators suppress even the most basic of physiologic processes. Breathing rates decrease from 40 breaths/minute to less than 1 breath/min as they decrease body temperature from 37 °C to ambient. Nevertheless, after months of hibernation, these incredible mammals emerge from torpor unscathed. This study was conducted to better understand the protective and possibly anti-inflammatory adaptations that hibernator lungs may use to prevent damage associated with entering and emerging from natural torpor. We postulated that the differential protein expression of soluble protein receptors (decoy receptors that sequester soluble ligands to inhibit signal transduction) would help identify inhibited inflammatory signaling pathways in metabolically suppressed lungs. Instead, the only two soluble receptors that responded to torpor were sVEGFR1 and sVEGFR2, two receptors whose full-length forms are bound by VEGF-A to regulate endothelial cell function and angiogenesis. Decreased sVEGFR1/2 correlated with increased total VEGFR2 protein levels. Maintained or increased levels of key γ-secretase subunits suggested that decreased sVEGFR1/2 protein levels were not due to decreased levels of intramembrane cleavage complex subunits. VEGF-A protein levels did not change, suggesting that hibernators may regulate VEGFR1/2 signaling at the level of the receptor instead of increasing relative ligand abundance. A panel of angiogenic factors used to identify biomarkers of angiogenesis showed a decrease in FGF-1 and an increase in BMP-9. Torpid lungs may use VEGF and BMP-9 signaling to balance angiogenesis and vascular stability, possibly through the activation of SMAD signaling for adaptive tissue remodeling.

## Introduction

In response to colder days and limited food abundance, some homeothermic endotherms use adaptive heterothermy (lowering body temperature from 37 °C to ambient 0–5 °C) and metabolic rate depression to save precious energy stores. Small mammalian hibernators decrease their heart rate from 200–300 beats per min (bpm) to 3–4 bpm and stifle their breathing for up to an hour only to take the few breaths they require to sustain basal metabolism (Boyer & Barnes, 1999; Storey & Storey, 2005). Hibernators also ensure proper tissue oxygenation by increasing cardiac contractile strength to pump viscous blood and decreasing the levels of circulating clotting factors and white blood cells to minimize blood clotting (Wang & Wolowyk, 1988; Bouma et al., 2013; Sahdo et al., 2013; Cooper et al., 2016). Yet, despite significant decreases in overall oxygen intake and body temperature, as well as possible oxidative insult from apnoic breathing, tissue damage has never been reported for hibernator lung.

At the molecular level, the relatively few studies on hibernator lung have almost exclusively focused on changes in cell membrane or surfactant lipid profiles, and pulmonary surfactant secretion pathways (Aloia, 1981; Langman, Orgeig & Daniels, 1996; Lopatko et al., 1999; Ormond, Daniels & Orgeig, 2001; Ormond, Orgeig & Daniels, 2003; Suri et al., 2012). However, the lung may be one of the few hibernator tissues that can invoke an inflammatory response, which makes it an important tissue to study in this context. There is evidence from three hibernating species (Syrian hamster, hedgehog, and 13-lined ground squirrel) that lung may modestly increase inflammation during torpor. Metabolically suppressed hamster lung shows elevated levels of pro-inflammatory proteins such as ICAM-1, VCAM-1, NFκB p65, and TGF-β, while hibernating hedgehogs increase the size of select leukocyte populations (namely neutrophils) (Talaei et al., 2012; Bohr, Brooks & Kurtz, 2014). Compared to summer ground squirrel lung torpid lung has less anti-inflammatory IL-10, SOCS1 and SOCS5 protein, and higher levels of pro-inflammatory markers such as leukocyte marker CD11b/c+ and the cell adhesion marker E-selectin (Bohr, Brooks & Kurtz, 2014). Thus, part of the adaptation to torpor could include a change in the profile of inflammatory proteins. This may seem counterproductive for lungs, considering it is energetically expensive to mount an inflammatory response, and chronic inflammatory responses can be quite damaging to the surrounding tissue. However, inflammation in torpid hamster lung has been shown to accompany adaptive changes in lung structure that could be reversible upon arousal (Talaei et al., 2011, 2012). Furthermore, studies on hibernators reveal that they have fewer circulating white blood cells; and they are slow to respond to lipopolysaccharide, T lymphocyte independent type-1 (TI-1) antigen, T lymphocyte independent type-2 (TI-2) antigens, and even complex mixtures of antigens from foreign animal blood injections (Bouma et al., 2013; Sahdo et al., 2013; Sieckmann et al., 2014). Thus, the enhanced pro-inflammatory response in lung during torpor is likely not part of the adaptive immune system, which is generally suppressed during hibernation, but instead could be an innate response to the changes that accompany metabolic suppression.

Due to the lack of evidence that lung is damaged during hibernation, we hypothesized that there must be a mechanism to detect and inhibit pro-inflammatory signaling, which can be damaging to the surrounding tissue when left unchecked. The aim of the current study was to characterize one possible mechanism, the role of soluble receptors in the regulation of pro-inflammatory cytokine signaling within a torpor bout of a model hibernator (*Ictidomys tridecemlineatus*). Soluble receptors are short variants of functioning membrane-bound receptors and they bind the same ligands but usually do not invoke a cellular response. These decoys remove ligands out of circulation and can either be created by mRNA splicing (e.g., IL-4R, EGFR, sgp130, or LIF-R) or by proteolytic cleavage (e.g., VEGFR, TNFR, IL-1R, or IL-2R) (Jones & Rose-John, 2002; Cai et al., 2011). They can also bind to membrane-bound receptors to prevent their heterodimerization or homodimerization and subsequent activation (Kendall, Wang & Thomas, 1996). In this study, 14 soluble cytokine receptors were assayed to determine which signaling pathways could be regulated by truncated decoy receptors, where most of these receptors controlled anti- and pro-inflammatory processes. Surprisingly, results of this analysis suggested that vascular endothelial growth factor receptor (VEGFR)-mediated angiogenic pathways were not inhibited by soluble receptors in the lung during torpor. Immunoblotting was then used to assess the relative abundance of VEGFR2, a potent regulator of angiogenesis, as well as crucial subunits of the gamma-secretase complex, a major regulator of soluble receptor protein levels. Downstream effects of angiogenic signaling were examined using two multiplex assays for more than a dozen angiogenic proteins involved in cross-talk with VEGFR-signaling pathways, including fibroblast growth factor (FGF), bone morphogenic protein (BMP), and transforming growth factor β (TGF-β). Our results, combined with current hibernator lung literature, suggested that angiogenic proteins could be involved in lung remodeling and inhibition of cell death during hibernation.

## Methods

### Animals

Animal experiments involving wild-captured thirteen-lined ground squirrels (*Ictidomys tridecemlineatus*) were performed at the National Institutes of Health (Bethesda, MD, USA) by the laboratory of Dr. J.M. Hallenback, as previously reported (Logan & Storey, 2016), in accordance with guidelines set by NINDS animal care and use committee (ACUC, #ASP 1223–05). Animals with sufficient food stores were transferred to an environmental chamber at 5 °C in constant darkness. Body temperature (T_b_) (monitored by a subcutaneously injected sensor chip), time, and respiration rate were used to determine sampling points. Ground squirrels were sacrificed during the winter months January and February when their natural torpor bouts are the deepest (lowest metabolic rate) and longest, approximately 10–14 days for various ground squirrel species (Geiser & Kenagy, 1988; Wang & Lee, 1996; Kisser & Goodwin, 2012). Euthermic in the cold room (EC) animals had a stable T_b_ (37 °C) at 5 °C and were able to enter torpor but had not done so for 3 days. Late torpor (LT) animals were continuously in deep torpor for at least 5 days with T_b_ values of 5–8 °C. Tissue samples were shipped to Carleton University on dry ice and were stored at −80 °C until use.

### Protein extraction

Protein extracts were prepared from frozen tissue samples as previously described (Rouble, Tessier & Storey, 2014). Protein concentration was determined using the Bradford assay (Bio-Rad, Cat#5000006) and samples were standardized to 20 μg/μL with Assay Buffer 2 (Millipore–Sigma; Cat#43-041) for both the soluble receptor and angiogenic multiplex assays. A protein concentration of 2.5 μg/μL was optimal for the TGFβ1-3 multiplex assay and standardization of the samples was performed with Sample Diluent (Millipore–Sigma, #LTGF-SD).

### Multiplex analysis

Multiplex panels were used to measure relative soluble receptor levels and angiogenic protein levels (Eve Technologies, University of Calgary; Human Soluble Cytokine Receptor Array 14-plex, #HDSCR14 and Human Angiogenesis Array & Growth Factor 17-plex Array, #HDAGP17). The protocol for each assay was performed by Eve Technologies personnel as instructed by the manufacturer. A Bradford protein assay was used to determine total protein concentration before loading the same amount of protein in each well. A multiplex assay for analytes TGF-β1-3 (Millipore–Sigma, MAP TGFß Magnetic Bead 3 Plex Kit, #TGFBMAG-64K-03) was performed in-house according to the manufacturer’s instructions. Multiplex data are expressed as median fluorescence intensity (MFI) ± SEM, *n* = 5 independent samples from different animals, except *n* = 4 was used for EGF (LT only) and TGFβ1-3 (EC only) analyses. Signals below the no-protein control baseline signal were not used in quantification.

### Western blotting

Western blotting was performed as previously described (Logan & Storey, 2018). Protein extracts combined 1:1 with 2X sodium dodecyl sulfate (SDS) buffer (100 mM Tris base, 4% w/v SDS, 20% v/v glycerol. 0.2% w/v bromophenol blue and 10% v/v beta-mercaptoethanol) were boiled and had a final sample concentration of 2 µg/µL. Cell Signaling primary antibodies for nicastrin (Cat#5665), presenilin 2 (Cat#9979) and VEGFR2 (Cat#2479) were diluted 1:1000 v/v in TBST (50 mM Tris–HCl, 150 mM NaCl, 0.05% v/v Tween-20, pH 6.8). HRP-linked anti-rabbit goat IgG secondary antibody (BioShop, Cat#APA007P.2) was diluted 1:4000 v/v in TBST. Bands were visualized by enhanced chemiluminescence. PVDF membranes stained with Coomassie Blue (0.25% w/v Coomassie brilliant blue, 7.5% v/v acetic acid, 50% methanol) were used as a protein-loading control for western blotting. Chemiluminescent protein band signal was divided by a Coomassie-stained region of the same lane (Eaton et al., 2013), in an area away from the quantified protein target, where the band density did not differ between control and hibernating states. Data (*n* = 4) are expressed as mean band density ± SEM, relative to EC values. RBioplot was used to analyze data with a Student’s *t*-test, where *p* < 0.05 represents statistical significance, and make the graphs (Zhang & Storey, 2016).

## Results

### Relative protein levels of soluble receptors in hibernator lung

**Figure 1:**
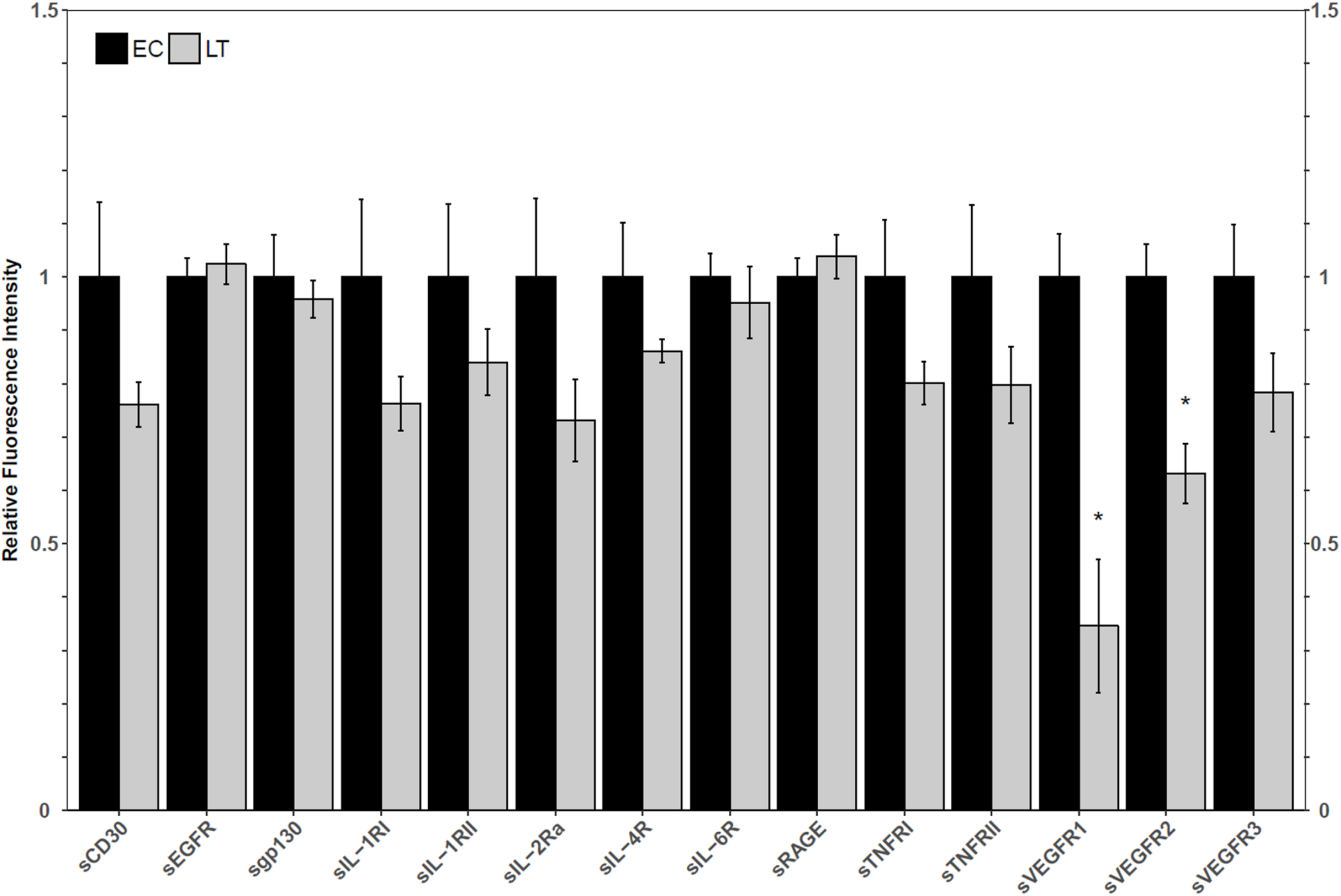
Relative total protein levels of soluble receptors in the lung tissue of euthermic in the cold room control (EC) and late torpid (LT) ground squirrels. Histogram showing relative mean protein levels (± S.E.M., *n* = 5 independent protein isolations from different animals). Where the Student’s *t*-test (*p* < 0.05) yielded statistical significance, an asterisk is shown above the LT bar.

Using a high-throughput approach, the relative fluorescence intensity levels of 14 soluble receptors were compared between lung from euthermic control (EC) and hibernating (LT) ground squirrel lung. The protein levels of sVEGFR1 and sVEGFR2 decreased to 35 ± 14% and 63 ± 6% of the EC level during LT (Fig. 1).

### Immunoblot analysis of VEGFR2 and gamma secretase complex subunits

**Figure 2:**
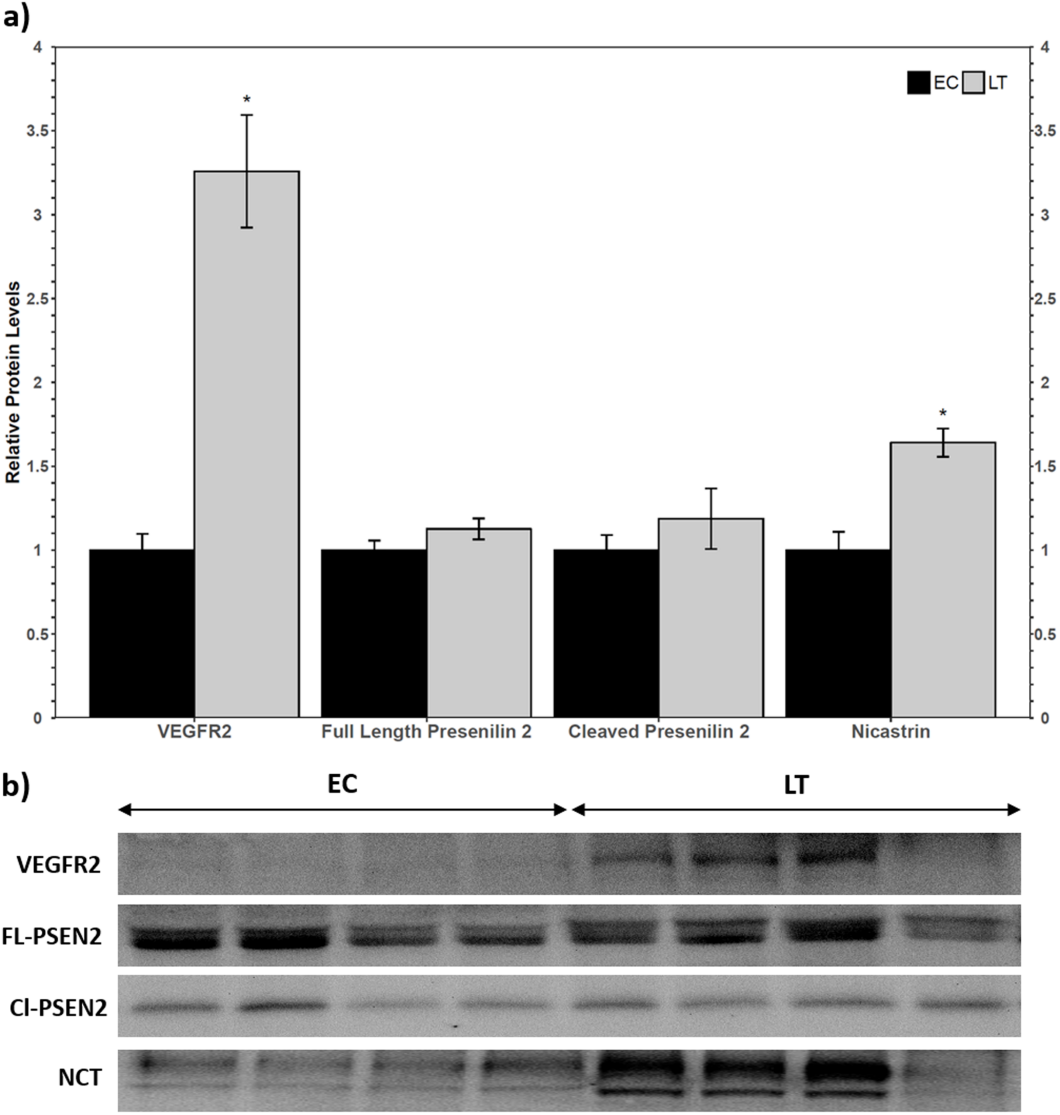
Relative total protein levels of vascular endothelial growth factor receptor 2 (VEGFR2) and gamma secretase subunits presenilin 2 and nicastrin in the lung of control (EC) and torpid (LT) ground squirrels. (A) Histogram showing relative mean protein levels of VEGFR2, presenilin 2, and nicastrin (± S.E.M., *n* = 4 independent protein isolations from different animals). (B) Representative western blots for certain torpor-arousal time points. An asterisk above the histogram LT bar indicates statistical significant differences between sample points (*p* < 0.05).

Western blotting of VEGFR2 identified a single band above the 175 kDa molecular weight marker, indicating that the full length VEGFR2 protein was detected and not the lower molecular weight soluble variant. The relative levels of VEGFR2 increased during torpor to 3.3 ± 0.3-fold the EC level (Fig. 2a). Two biomarkers of the gamma secretase complex were also assessed for their relative protein levels during torpor compared to euthermia. Presenilin 2 can be proteolytically cleaved into an active form that can be detected at 23 kDa, where a strong band was present for ground squirrel. The band representing full length presenilin 2 was detected close to the predicted molecular weight of 50 kDa. Euthermic and hibernating animals had similar levels of full-length and cleaved presenilin 2 (Fig. 2). According to the antibody manufacturer and published literature, nicastrin appears between 100 and 140 kDa. The bands at 140 kDa and 150 kDa were quantified together based on previous reports of nicastrin appearing as a doublet high above the predicted molecular weight. When considering the band densities of both nicastrin bands, the relative protein levels of nicastrin increased 1.6 ± 0.08-fold the EC levels during LT (Fig. 2).

### Relative protein levels of angiogenic factors and TGF-β 1, 2, and 3 in hibernator lung

**Figure 3:**
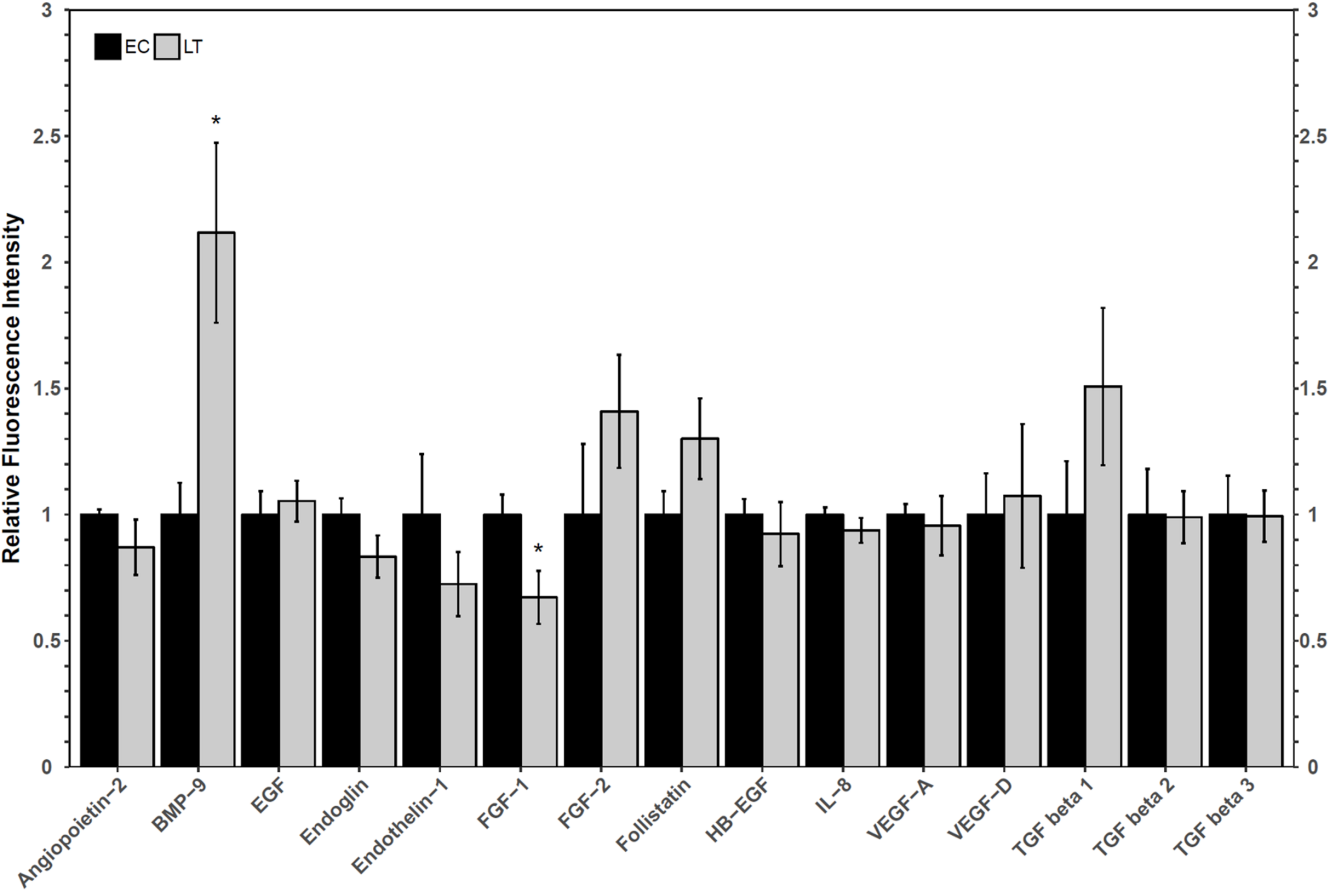
Relative total protein levels of angiogenic markers and TGF-β1-3 proteins in the lung tissue of euthermic in the cold room control (EC) and late torpid (LT) ground squirrels. Histogram showing relative mean protein levels (± S.E.M., *n* = 5 independent protein isolations from different animals, with an *n* = 4 being used for the LT group of EGF analysis and for the EC group of the three TGF-β proteins). Where the Student’s *t*-test (*p* < 0.05) yielded statistical significance, an asterisk is shown above the LT bar.

A Luminex assay was used to quantify the relative protein levels of 12 angiogenic proteins to determine how hibernators regulate angiogenic protein expression in torpor. The analysis revealed an increase in hibernating BMP-9 protein levels to 2.1 ± 0.4-fold, relative to control levels. Furthermore, FGF-1 levels decreased during LT to 67 ± 12% of EC (Fig. 3). The protein levels of the other angiogenic factors did not change relative to the control. Similarly, there were no significant differences in the relative protein levels of any of the three TGF-β proteins assessed (Fig. 3).

## Discussion

Hibernators have long been regarded as excellent model organisms capable of resisting tissue damage in the face of unavoidable cell stress. The lung is of interest for its importance in delivering oxygen to the whole body. The current study aimed to identify in what capacity soluble receptors may be involved in the regulation of inflammatory signaling during torpor. Decoy receptors are typically shorter and do not span the cell membrane but can bind the same ligands as transmembrane receptors without inducing a signaling response. Thus, the levels of decoy receptors should inversely correlate with the activity of any particular transmembrane receptor signaling pathway. The absence of a change in decoy receptor levels does not rule out the involvement of the pathways that signal through the full-length receptors, but it suggests that soluble receptors may not regulate these pathways. For this reason, the following analysis chose to focus solely on the soluble receptors that exhibited changes between EC and LT. Through a multiplex analysis of relative soluble receptor levels, significant decreases in the total protein levels of sVEGFR1 and sVEGFR2 were identified, which suggested the ground squirrel lung may actively regulate the VEGF signaling pathway during torpor (Fig. 1). VEGFR1 and VEGFR2 both accept VEGF-A as a ligand and trigger angiogenesis, but VEGFR3, whose soluble variant did not decrease during torpor relative to the EC, accepts VEGF-C and VEGF-D as ligands to initiate lymphangiogenesis (Shibuya, 2013). Since the soluble form of a receptor functions as a decoy for the membrane-bound receptor, VEGFR1 and VEGFR2-mediated signaling could be upregulated during torpor.

VEGFR2 is a more potent inducer of VEGF-A signaling than VEGFR1 (and in some species, it is the only receptor required for angiogenesis) (Hornig & Weich, 1999; Yamaguchi, Iwata & Shibuya, 2002), so it was used as a biomarker for VEGF-A signaling in hibernator lung. Western blot analysis revealed an increase in VEGFR2 protein levels (Fig. 2), which suggested that VEGF-mediated signaling could indeed be upregulated during torpor. VEGF-A levels remained constant relative to the EC (Fig. 3), so decreased sVEGFR1/sVEGFR2 and increased VEGFR2 levels suggest that angiogenic signaling is regulated at the level of the receptor instead of by relative ligand abundance. The γ-secretase complex is responsible for the intramembrane cleavage of over 60 receptors, including VEGFR1 and VEGFR2 (Boulton, Cai & Grant, 2008; Cai et al., 2011; De Strooper, Iwatsubo & Wolfe, 2012). To confirm that the levels of sVEGFR1 and sVEGFR2 were not downregulated as a result of decreases in the relative abundance of γ-secretase protease complex subunits, the relative protein levels of two important γ-secretase biomarkers were assessed via immunoblotting. The relative levels of the catalytic subunit, presenilin 2, did not change and the levels of nicastrin increased. Nicastrin is required for the recognition of substrates targeted for cleavage (Yang et al., 2002; Cai et al., 2011). Mature nicastrin is both heavily glycosylated and *S*-palmitoylated in order to facilitate interactions with the catalytic subunit presenilin, and was observed as a doublet high above the expected molecular weight (78 kDa) of the unmodified protein (Yang et al., 2002; Cheng et al., 2009). Nicastrin from HEK293 and MDCK cells was also observed at ~120 kDa and 110 kDa on a western blot and nicastrin from mouse brain cells was seen at ~125 kDa and 120 kDa, suggesting that nicastrin is regulated by glycosylation at varying degrees in different cell types (Yu et al., 2000; Yang et al., 2002). In this study, the heavier nicastrin band was denser than the lower band of the doublet, implying that there is generally more mature nicastrin than partially post-translationally modified nicastrin in ground squirrel lung (Fig. 2b). Increases in nicastrin and maintained presenilin 2 levels during torpor suggested that ground squirrels may employ differential proteolysis of membrane receptors as a mechanism of regulating signaling pathway activity. Thus, the decrease in sVEGFR levels may be an active form of regulation of VEGFR signaling instead of a result of fewer γ-secretase complexes. For instance, there could be fewer sVEGFR1/2 because the splice variants for these short receptors are not preferred over the long form during metabolic suppression (Hornig & Weich, 1999; Fellows, Mierke & Nichols, 2013). The γ-secretase complex can cleave over 60 intramembrane substrates (De Strooper, 2005; De Strooper, Iwatsubo & Wolfe, 2012), so further analysis will be warranted to characterize its complete regulatory role during hibernation. Together, these results suggest that the VEGFR1 and VEGFR2 signaling pathways are less inhibited by decoy receptors and that VEGFR2 may be upregulated to augment VEGF signaling during torpor.

Oxidative stress, which is possible in a lung environment in which an animal breathes only intermittently, could promote angiogenesis through hypoxia-inducible factor-VEGF mediated mechanisms as well as VEGF-independent mechanisms (Kim & Byzova, 2014). Importantly, VEGF pathways, especially those involving VEGFR2, can promote apoptosis and angiogenesis through angiogenic factors such as FGF and TGF-β proteins (Ferrari et al., 2009). A multiplex analysis of angiogenic factors was performed to help determine the possible downstream implications of VEGFR signaling in the lung of hibernating ground squirrels (Fig. 3). Only two angiogenic factors varied in their total protein levels: FGF-1 and BMP-9. Since both proteins influence TGF-β signaling, a multiplex analysis of three TGF-β proteins was performed to further characterize angiogenic pathway regulation in a metabolically suppressed mammal.

FGF-1 is a pro-angiogenic factor whose relative protein levels decreased during torpor, suggesting a decrease in angiogenesis in lung during torpor. In human and rat lung cells, FGF-1 and TGF-β show opposite protein expression patterns, and the overexpression of FGF-1 can decrease TGF-β1 signaling pathways (Ramos et al., 2010; Shimbori et al., 2016). FGF-1 can regulate fibroblast apoptosis as well as cell structure (by increasing collagenase 1 and decreasing collagen type 1 levels), making it an important factor in the regulation of angiogenesis and tissue repair (Ramos et al., 2010; Shimbori et al., 2016). The decrease in FGF-1 observed in hibernating ground squirrel lung is consistent with reports of increases in TGF-β1, collagen, and smooth muscle actin levels in early torpid Syrian hamsters (Talaei et al., 2011). Indeed, TGF-β1, 2, and 3, levels were maintained at euthermic levels during torpor in 13-lined ground squirrel lung (Fig. 3). It is possible that hibernating ground squirrels may not need to upregulate TGF-β levels, but instead, that euthermic TGF levels could be sufficient to help coordinate angiogenic signaling in the torpid lung. Upon binding to a receptor, FGF-1 activates a variety of signaling pathways that can ultimately activate the expression of matrix metalloproteinase proteins (MMPs), enzymes that regulate extracellular matrix composition and aid in the remodeling of lung structure (Ramos et al., 2010; Raju et al., 2014; Ornitz & Itoh, 2015). A decrease in MMP activity was observed in hibernating hamster lung (Talaei et al., 2012), suggesting that the decrease in FGF-1 in 13-lined ground squirrel lung (Fig. 3) could also be associated with a decrease in MMP activity in obligate hibernators. The Syrian hamster also increases the levels of intercellular and vascular cell adhesion molecule 1 (ICAM-1 and VCAM-1), pro-inflammatory NF-κB p65 subunit, angiotensin converting enzyme, and decreases caveolin-1 and EGFR, which suggests overall lung structure changes throughout torpor bouts (Talaei et al., 2011, 2012). Thus, if the obligately hibernating 13-lined ground squirrel lung and the facultatively hibernating Syrian hamster lung adapt similarly to torpor, it’s possible that a downregulation of FGF-1 could play an important role in regulating the signaling cascades that promote adaptive changes in lung architecture during torpor.

An increase in BMP-9 levels could also help regulate lung structure and function during deep torpor. Ground squirrels increased BMP-9 protein levels during torpor but TGF-β proteins 1–3 did not change compared to the EC (Fig. 3). These results are similar to those reported for the Syrian hamster lung, in which TGF-β1 protein levels were high during early torpor and early arousal but were no different from the EC during deep torpor (Talaei et al., 2011). These results, in combination with the analysis of collagen and angiotensin converting enzyme levels, suggest that most lung reorganization occurs before the hibernator enters complete torpor or as it arouses from torpor (Talaei et al., 2011). Though only BMP-9 increased during torpor, both BMP-9 and TGF-β1 regulate vasculature makeup by signaling through Type II receptors (TGF-β receptor type II, BMP receptor type II, and activin receptor type II). Type II receptors activate Type I receptors such as activin receptor-like kinases to induce SMAD phosphorylation and subsequent gene transcription (Jin, Kaluza & Jakobsson, 2014; Jonker, 2014; Nickel, Ten Dijke & Mueller, 2018; Rol et al., 2018). Furthermore, BMP-9 may more strongly induce SMAD signaling than TGF-β1 by binding to the same receptors with higher affinity (Jin, Kaluza & Jakobsson, 2014). It has been proposed that BMP signaling primarily activates SMAD1/5/8-mediated cell proliferation and angiogenesis, whereas TGF-β signaling activates pro-fibrotic SMAD2/3 (Horbelt, Denkis & Knaus, 2012; Jonker, 2014; Myllärniemi et al., 2014; Long et al., 2015). Thus, a relative increase in BMP-9 protein vs. unchanging TGF-β protein levels suggest that SMAD1/5/8 signaling could be prioritized over SMAD2/3 signaling during torpor in the lung. This notion is consistent with a recent study performed on rats with hyperoxia-induced bronchopulmonary dysplasia, in which increased BMP-9 decreased lung fibrosis and had anti-inflammatory properties, yet did not reduce vascular remodeling (Chen et al., 2017). Future transcription factor studies detailing the post-translational activation of SMADs, their subcellular localization, and DNA binding ability during torpor is required to confirm this suspicion.

Of note, many of the angiogenic factors that were not upregulated have roles that would be maladaptive for hibernating lung. Some lines of evidence suggest that FGF-2 and TGF-β1 are overexpressed in fibrotic tissue, while others postulate that FGF-2 is only upregulated in response to lung injury (Guzy et al., 2015; Chen et al., 2016). FGF-2 functions in concert with heparin-binding epidermal growth factor (HB-EGF) and EGF to downregulate the expression of extracellular matrix proteins such as elastin, which are important in lung tissue repair processes (Liu et al., 2003). Perhaps FGF-2 protein levels did not increase in hibernator lung because BMP-9 enhances the stability of vasculature by inhibiting angiogenic factors like FGF-2 (Scharpfenecker et al., 2007). Using antibodies designed for human protein samples with rodent lung samples could increase the rate of false negatives by decreasing antibody binding to the epitope. However, most inflammatory and angiogenic proteins are highly conserved with high sequence identity between 13-lined ground squirrels and humans. Notably, all proteins that were differentially regulated during torpor had high homology with human protein sequence (>85% sequence identity), so false positives are unlikely, and even some proteins that didn’t change expression like follistatin and sGP130 have very high sequence identities (98.5% and 89.53%, respectively). Though appropriate controls were used to control for false negatives (e.g., loading sufficient protein amount, no protein blank), it is important to consider that some ground squirrel soluble receptors or angiogenic proteins that were reported to not change relative protein levels may indeed be differentially expressed during torpor in lung, but these changes are not detected by suboptimal antibodies.

Overall, angiogenic proteins implicated in tissue injury and fibrosis (TGF-β1, FGF-2, EGF, and HB-EGF) were maintained at euthermic levels during torpor, so it is unlikely that there is any lasting lung fibrosis or injury in hibernating animals. However, these proteins may have important roles in lung remodeling at other points of the torpor-arousal cycle, such as TGF-β in Syrian hamsters (Talaei et al., 2011). Another angiogenic factor the functions of which would not be adaptive in torpid ground squirrel lung is endothelin-1. Endothelin-1 maintains vascular tone when maintained at basal levels, but when its protein levels increase it inhibits K_ATP_ channels, resulting in vasoconstriction (Gaur et al., 2018). This would be maladaptive in the hibernating ground squirrel, as cold, viscous blood would have a harder time navigating through the circulatory system of the lung, making it increasingly difficult to transport oxygen to the rest of the body. Angiopoietin-2 protein levels also did not change during LT. Angiopoietin-2 functions as an inhibitor of angiopoietin-1 signaling, and in this way, angiopoietin-2 promotes inflammation and disrupts vascular maintenance. Indeed, its overexpression is associated with increased pulmonary vasculature permeability and lung injury (Parikh et al., 2006). IL-8, another angiogenic factor that was not differentially expressed in hibernating lung, lies downstream of angiopoetin-2, suggesting that basal levels of angiopoietin-2 do not induce inflammatory signaling in the hibernator lung. Thus, preventing the overexpression of angiopoietin-2 during torpor would be ideal, since angiopoietin-1 signaling could be important for lung remodeling and inhibiting inflammation.

Unlike many of the angiogenic factors that were not differentially regulated during torpor, BMP-9 probably exerts protective functions during torpor. BMP-9 can prevent apoptosis and pathological vascular proliferation to maintain vascular stability (Scharpfenecker et al., 2007; Shao et al., 2009; Long et al., 2015). Furthermore, both BMP-9 and TGF-β1 pathways can enhance angiogenesis by increasing the gene expression of VEGFR1, VEGFR2 and their ligand, VEGF-A (Ferrari et al., 2009; Jin, Kaluza & Jakobsson, 2014). Contradictory reports of BMP-9 inhibiting VEGFR2 signaling by increasing the expression of DNA binding inhibitors ID1 and ID3 emphasize that angiogenic signaling is highly dependent on the cellular context and species of study (Suzuki et al., 2010; Kim et al., 2012). However, based on the protein analysis herein, it’s possible that hibernators use BMP-9 signaling to regulate angiogenesis via the expression of VEGFR2. Overall, increased BMP-9 in the hibernator lung could play a key role in the regulation of lung architecture and function during torpor, whether it be through the inhibition of tissue fibrosis, the promotion of endothelial cell activation and angiogenesis, or the inhibition of inflammatory processes.

## Conclusion

Despite a change to apnoic breathing and body temperature approaching ambient, the lungs of hibernating mammals do not show signs of damage due to oxidative stress or low T_b_. The few studies to date suggest hibernators make use of coordinated changes in protein profile and enzyme activity that promote tissue remodeling. The current study is suggestive of a previously unexplored mechanism whereby ground squirrel lung enhances VEGF signaling. Soluble decoy receptors (VEGFR1 and VEGFR2) are suppressed and the total protein levels of VEGFR2, a highly potent VEGF-A signal transducer, increase. Analysis of nicastrin and presenilin 2 total protein levels demonstrated that the γ-secretase complex is likely not inhibited during torpor, but instead, that the decrease in sVEGFR1 and sVEGFR2 levels could be the result of a decrease in the cleavage of VEGFRs. The total protein levels of only two angiogenic factors changed during torpor: FGF-1 decreased and BMP-9 increased. Each exerts an opposite effect on TGF-β, which is elevated at several points of the torpor-arousal cycle in hibernating hamsters, but is maintained at euthermic levels during LT in two hibernating species. The other angiogenic factors that were maintained during torpor are commonly upregulated in injured lung and have pro-fibrotic functions, but the literature provides no evidence of either phenomenon in hibernator lung. Thus, hibernators may regulate angiogenic factors in the lung to promote adaptive tissue remodeling and prevent lung fibrosis. Future studies should focus on SMAD signaling as a potentially key transcription factor in the modulation of hibernating lung phenotype. Overall, this study has identified VEGF and BMP-9/TGF-β signaling cascades as potentially important modulators of lung plasticity in an extreme mammalian model.

## Additional Information and Declarations

### Field Study Permissions

The following information was supplied relating to field study approvals (i.e., approving body and any reference numbers): Ground squirrels were wild captured by United States Department of Agriculture (USDA) licensed trappers (TLS Research, Bloomingdale, IL, USA) annually in August and transported to the National Institutes of Health (Bethesda, MD, USA) where experiments were performd by the laboratory of Dr. J.M. Hallenback.

### Data Availability

The following information was supplied regarding data availability: Multiplex data is available at Figshare: Logan, Samantha (2019): Lung Angiogenesis Multiplex Assays. figshare. Dataset. https://doi.org/10.6084/m9.figshare.9226517.v1. Western blot images that can be accessed as JPEGs (or using GeneSnap or GeneTools software that can be downloaded online) are available at Figshare: Logan, Samantha (2019): Lung Angiogenesis Western Blots. figshare. Dataset. https://doi.org/10.6084/m9.figshare.9226643.v1.
